# Accuracy assessments of gridded precipitation data: A case study from LTAR Texas Gulf

**DOI:** 10.1002/jeq2.70236

**Published:** 2026-08-20

**Authors:** Merilynn C. Hirsch‐Schantz, Douglas R. Smith, R. Daren Harmel, Kabindra Adhikari, Kelly R. Thorp, Stuart P. Hardegree, Andrew Fullhart, John T. Abatzoglou, Javier M. Osorio Leyton, Roger Sheley, Aaron Hird

**Affiliations:** ^1^ USDA‐ARS, Grassland Soil and Water Research Temple Texas USA; ^2^ USDA‐ARS, Center for Agricultural Resources Research Center Fort Collins Colorado USA; ^3^ USDA‐ARS, Northwest Watershed Research Center Boise Idaho USA; ^4^ Department of Natural Resources and the Environment University of Arizona Tucson Arizona USA; ^5^ Sierra Nevada Research Institute University of California‐Merced Merced California USA; ^6^ Rangeland, Wildlife & Fisheries Department Texas A&M AgriLife Blackland Research and Extension Center Temple Texas USA; ^7^ USDA‐ARS, Range and Meadow Forage Management Research Unit Burns Oregon USA; ^8^ USDA Natural Resources Conservation Service (NRCS), Conservation Effects Assessment Program (CEAP) Lincoln Nebraska USA

## Abstract

Extreme meteorological events contribute to 80% of agroecosystem loss indemnities. Daily, seasonal, and annual precipitation dynamics also affect plant production, erosion, sediment and nutrient loading, and soil health across agroecosystems, largely depending on precipitation timing, amount, frequency, and intensity. Gridded meteorological data have been publicly available since the mid‐2000s and used to assess spatiotemporal precipitation dynamics. The accuracy of gridded compared to site‐level precipitation is, however, rarely evaluated because most long‐term meteorological data are already calculated into gridded databases. At the Long‐Term Agroecosystem Network Texas Gulf site in central Texas, however, a nearly 90‐year precipitation record exists from a suite of 15 on‐site rain gauges that have not been used in gridded dataset development. The objective of this study was to evaluate the accuracy of historical precipitation acquired from 15 on‐site monitoring stations with the common gridded databases of the Parameter‐Elevation Regressions on Independent Slopes Model (PRISM), the Daily Surface Weather and Climatological Summaries (DayMET), and the Gridded Surface Meteorological Dataset (GridMET). Our findings suggest precipitation is highly variable spatiotemporally. Annual and seasonal gridded data from all sources were significantly correlated with on‐site weather station precipitation, but GridMET produced the strongest correlations with on‐site data. Across time, the accuracy of gridded precipitation data improved, especially between 1980 and 2000 decades. Extreme daily precipitation events acquired from gridded data sources, however, were poorly correlated with actual precipitation at rain gauge sites. These results suggest that gridded data can be helpful for long‐term management planning but also showcase a limited utility of gridded data for monitoring assessments, especially as they relate extreme precipitation to erosion, crop loss, and insurance indemnities.

## Core Ideas

- Precipitation abundance, timing, and intensity are primary drivers of agroecosystem production, conservation, and management.
- Many gridded climate products are available, but their accuracy is rarely evaluated.
- Average monthly and annual precipitation was accurate for gridded data, but daily data were skewed.
- For this site, the Gridded Surface Meteorological Dataset (GridMET) produced the most accurate data, followed by the Daily Surface Weather and Climatological Summaries (DayMET) and the Parameter‐Elevation Regressions on Independent Slopes Model (PRISM).
- Gridded data were not well suited for assessing small spatiotemporal precipitation dynamics
- Monitoring extreme meteorological events should continue at the site level, especially as it relates to erosion and insurance.

## Plain Language Summary

Precipitation directly affects plant production, erosion, and soil health across agroecosystems depending on amount, timing, and intensity. Gridded precipitation data can assist in understanding these dynamics across space and time. There are, however, multiple gridded data products available, and their quality is rarely compared because precipitation collected from most monitoring stations is currently used in gridded data production. In central Texas, a precipitation record exists that is not used in gridded dataset development. From these data, we found that historical precipitation is highly variable across space and time. While all gridded data were significantly correlated to on‐site weather station precipitation data across annual and seasonal scales, the Gridded Surface Meteorological Dataset had the greatest accuracy. Extreme daily precipitation events, however, were not well characterized in gridded data, which limits the utility of gridded data for some erosion and watershed models and insurance monitoring.

**Abbreviations**
CONUS: contiguous United States
DayMET: Daily Surface Weather and Climatological Summaries
ENSO: El Niño Southern Oscillation
GridMET: Gridded Surface Meteorological Dataset
LTAR: Long‐Term Agroecosystem Research
PRISM: Parameter‐Elevation Regressions on Independent Slopes Model
RMSE: root mean squared error
WXYRD: weather yard rain gauge station

## 1 INTRODUCTION

Precipitation is a primary driver of plant production, hydrologic processes, sediment and nutrient loading, soil health, and erosion in agroecosystems (Chen et al., 2019; Huxman et al., 2004; Murphy et al., 2015; Waller et al., 2021). Quantifying the spatial and temporal distribution of precipitation is vital for water resource management, forage availability calculations for stocking rates, fuel load estimates, risk mitigation, and restoration management planning decisions. The interactions between available water and meteorological patterns are becoming even more important as shifts in regional temperature and precipitation patterns are resulting in a greater frequency of meteorological extremes, such as heatwaves, intense drought, and heavy, short‐duration precipitation events (Basara et al., 2013; Flanagan & Mahmood, 2023; Flanagan et al., 2017; Gherardi & Sala, 2020).

For hundreds of years, precipitation and temperature have been collected at point‐scale locations (Gebremichael, 2010; Segovia‐Cardozo et al., 2023). Precipitation is currently being monitored at more than 11,000 ground‐based weather stations via a network of standard 8″ non‐recording precipitation gauges associated with the US climate station network monitored by the National Weather Service (WMO [World Meteorological Organization], 2025). There are, however, limitations in relying on ground‐based precipitation data largely due to variability in data collection methods and location effects at weather monitoring stations. For example, many stations rely on tipping bucket sensors, which are known to underestimate the total precipitation due to wind, evaporation, splashing, and wetting phenomena (Duchon & Biddle, 2010; Lanza & Cauteruccio, 2022; Segovia‐Cardozo et al., 2023; Shedekar et al., 2016). Many weather monitoring stations are also located in cities and/or airports (WMO, 2025), which can have temperature and precipitation bias associated with localized warming from anthropogenic development (Sakthivel & Sengupta, 2025; Stewart & Oke, in press). Station density is also a problem for producing accurate precipitation estimates across space, especially in rural regions where few ground‐based weather stations exist and stations are often separated by many miles of complex topography (Essou et al., 2017; Ward et al., 2011). Station density and coverage can also change over time as old stations are decommissioned or new stations added, which can lead to biases, especially for remote areas (Sun et al., 2016).

To overcome station–data spatial limitations, Daly et al. (2008) used a weighted average calculation to interpolate data from ground‐based weather stations and calculate weather and climate metrics for equidistant (generally 0.8–4 km^2^) grid points spanning the continental United States. Other authors subsequently used similar methods to generate weather and climate data for agroecosystem management planning across space (e.g., Abatzoglou, 2013; Allen et al., 2021; Thornton et al., 2014).

Gridded climate datasets offer several advantages over ground‐based weather station data including ease of use, uniform spatial coverage, consistent coverage over time, and serially complete datasets (Essou et al., 2016). For these reasons, gridded weather and climate data are actively used in decision‐making tools and products, such as hydrologic models (Abbott et al., 1986; Arnold et al., 2012; Bergström, 1995), plant production assessments (Rigge et al., 2021; Robinson et al., 2019), and forecasting products (Hartman et al., 2020; Schantz et al., 2025, 2026). Gridded products and their calculations are often cross‐referenced for quality using historical weather and climate datasets and associated ground‐based monitoring stations; yet, there are still questions on the accuracy of these data. Accuracy assessments of gridded meteorological data, for example, have been limited given that there are few long‐term, quality‐controlled ground‐based monitoring sites that are not already used in the development of the gridded weather and climate datasets (Ray et al., 2022).

A key limitation to consider when using gridded weather data is that weather varies across small spatiotemporal scales, especially across complex terrain (Bradford et al., 2006; Hardegree et al., 2012). In rural areas, few long‐term quality‐controlled ground‐based monitoring stations exist, which can result in inaccurate estimates of weather and climate when calculated for fine grid scales (WMO, 2025). In a comprehensive Canadian study, Essou et al. (2017) identified that across most of a 316‐station watershed network, weather station density was less than one station per 1000 km^2^ and that many gridded data estimates were inaccurate due to the space between ground‐based monitoring stations. Gridding calculations can also result in data smoothing, which overestimates precipitation abundance at low amounts and underestimates precipitation at high amounts (Ensor & Robeson, 2008). Another limitation of gridded data as it relates to agriculture and natural resource monitoring is that precipitation timing and intensity are averaged from nearby stations, which limits the ability to determine the direct effects of precipitation at a point in time on sediment loss and nutrient loading into waterways. Ward et al. (2011), for example, found that gridded data were not well correlated with river discharge estimates in mountainous South American ecosystems and suggested relying on ground‐based measurements in these topographically diverse regions. As extreme meteorological events are the leading cause of agricultural insurance claims, accounting for approximately 80% of crop insurance indemnities (ERS [Economic Reserve Service], 2024), accurately quantifying extreme meteorological events spatially and temporally on production agriculture is important for sustaining agricultural production. Spatiotemporal evaluations of gridded data are needed to determine the accuracy and limitations of these datasets for use in agroecosystem models, decision support tools, and resource management planning.

**Core Ideas**

- Precipitation abundance, timing, and intensity are primary drivers of agroecosystem production, conservation, and management.
- Many gridded climate products are available, but their accuracy is rarely evaluated.
- Average monthly and annual precipitation was accurate for gridded data, but daily data were skewed.
- For this site, the Gridded Surface Meteorological Dataset (GridMET) produced the most accurate data, followed by the Daily Surface Weather and Climatological Summaries (DayMET) and the Parameter‐Elevation Regressions on Independent Slopes Model (PRISM).
- Gridded data were not well suited for assessing small spatiotemporal precipitation dynamics
- Monitoring extreme meteorological events should continue at the site level, especially as it relates to erosion and insurance.

The southern Great Plains provides a unique and critical region for testing the accuracy of gridded verses to rain gauge precipitation data. This region has a long history of production agriculture, but great swaths of agricultural land are actively being urbanized (Londe et al., 2022). Moreover, conventional management strategies like annual tillage are still commonly used throughout most of this region, thus increasing erosion potential (Garbrecht et al., 2015; Hill et al., 1944). The climate of the southern Great Plains is also dynamic, and with many years of extreme precipitation patterns (i.e., deluge or drought) and fewer years with moderate and evenly distributed precipitation (Harmel et al., 2003).

The Texas Gulf (a research site associated with the USDA‐LTAR network) Long‐Term Agroecosystem Research (LTAR) site in Riesel, TX, has a nearly 90‐year precipitation record which could provide a useful dataset for quality assurance tests of gridded precipitation data. This site has experienced extremely dynamic precipitation and is in a rural and agriculturally important area (Harmel et al., 2003). Because this precipitation dataset was not used for gridded data creation and development, there is a unique opportunity to compare long‐term precipitation data from on‐site stations with gridded products at multiple spatiotemporal scales. The objective of this study was to quantify the differences between historical precipitation data acquired from 15 on‐site ground‐based monitoring stations and data from PRISM (Parameter‐Elevation Regressions on Independent Slopes Model), DayMET (Daily Surface Weather and Climatological Summaries), and GridMET (Gridded Surface Meteorological Dataset) databases at the LTAR Texas Gulf, Riesel Watersheds in Riesel, TX. We hypothesized that differences between rain gauge and gridded data sources would (1) vary by gridded data source and (2) vary by time, where gridded data accuracy would vary both annually and seasonally, and (3) extreme precipitation events would not be well captured using gridded data.

## 2 MATERIALS AND METHODS

### 2.1 Study site

**FIGURE 1:**
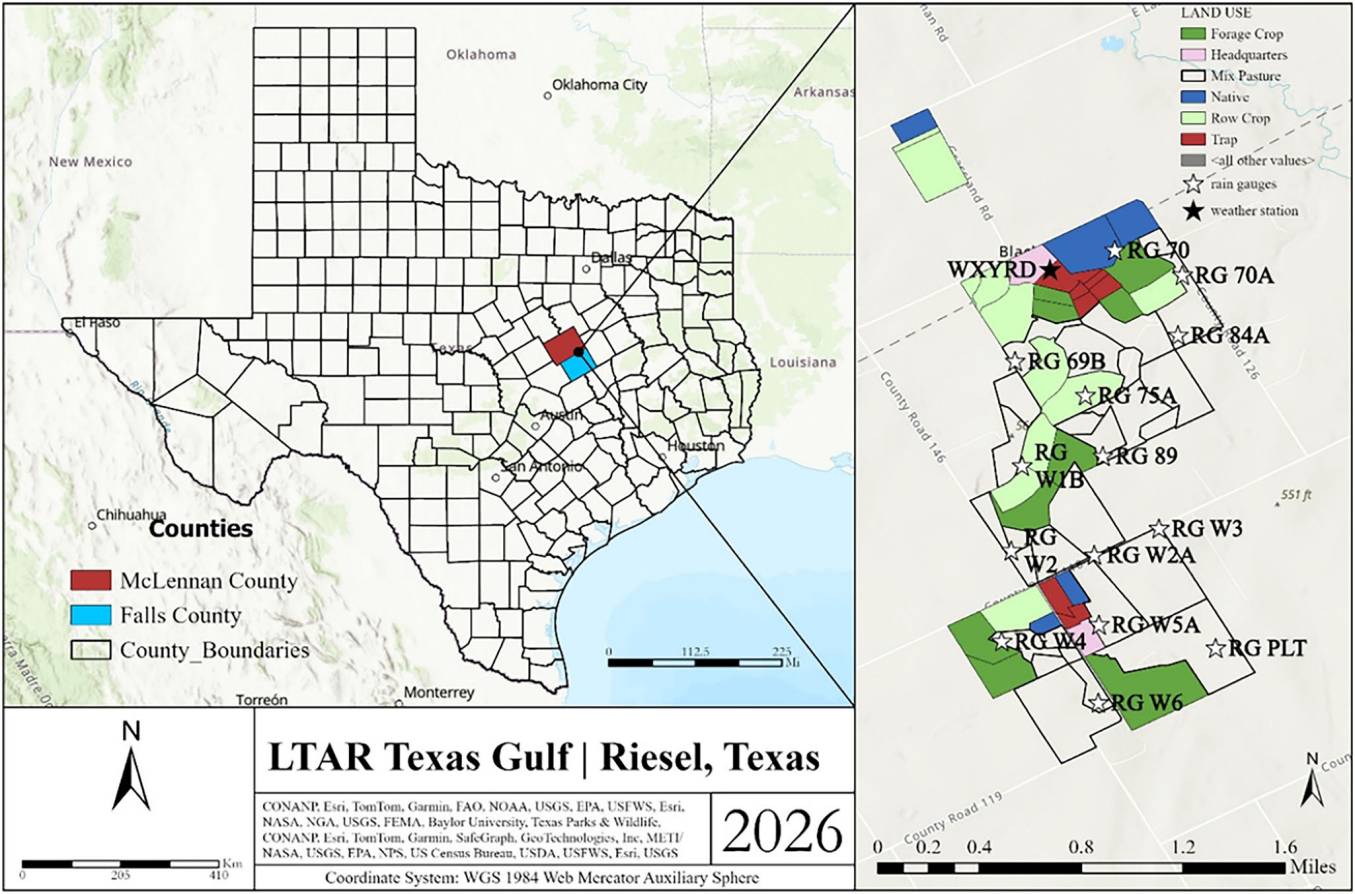
LTAR (Long‐Term Agroecosystem Research) Texas Gulf study site location and associated agroecosystem land use. This site falls on the border of two counties, McLennan and Falls, as indicated in the larger map and by the dashed line on the zoomed‐in version, where McLennan County is north of the dashed line, and Falls County is south. Rain gauge sites are indicated with white stars, and the weather station (WXYRD [weather yard rain gauge station]), which measures the full suite of weather variables, is indicated with the black star. RG, rain gauge.

This study was conducted at the LTAR Texas Gulf site at the USDA–ARS (United States Department of Agriculture–Agricultural Research Service) Grassland Soil and Water Research Laboratory (GSWRL) watershed facility near Riesel, TX (Figure 1). This site contains 340 ha of federally owned and operated land, primarily used for grazing livestock, hay production, and row crop production of corn and cotton. The GSWRL was established in 1937 as a Soil and Water Conservation Research site on the 2372 ha Brushy Creek watershed near Riesel, TX, because of its central location in the 4.45 million ha Texas Blackland Prairie and due to its importance in understanding agricultural management practices on soil loss, especially following the Dust Bowl (Harmel et al., 2007). Across this site, Houston Black clay (very‐fine, smectitic, thermic Oxyaquic Hapluderts) is the most extensive soil series, which is noted for its strong shrink/swell properties.

**FIGURE 2:**
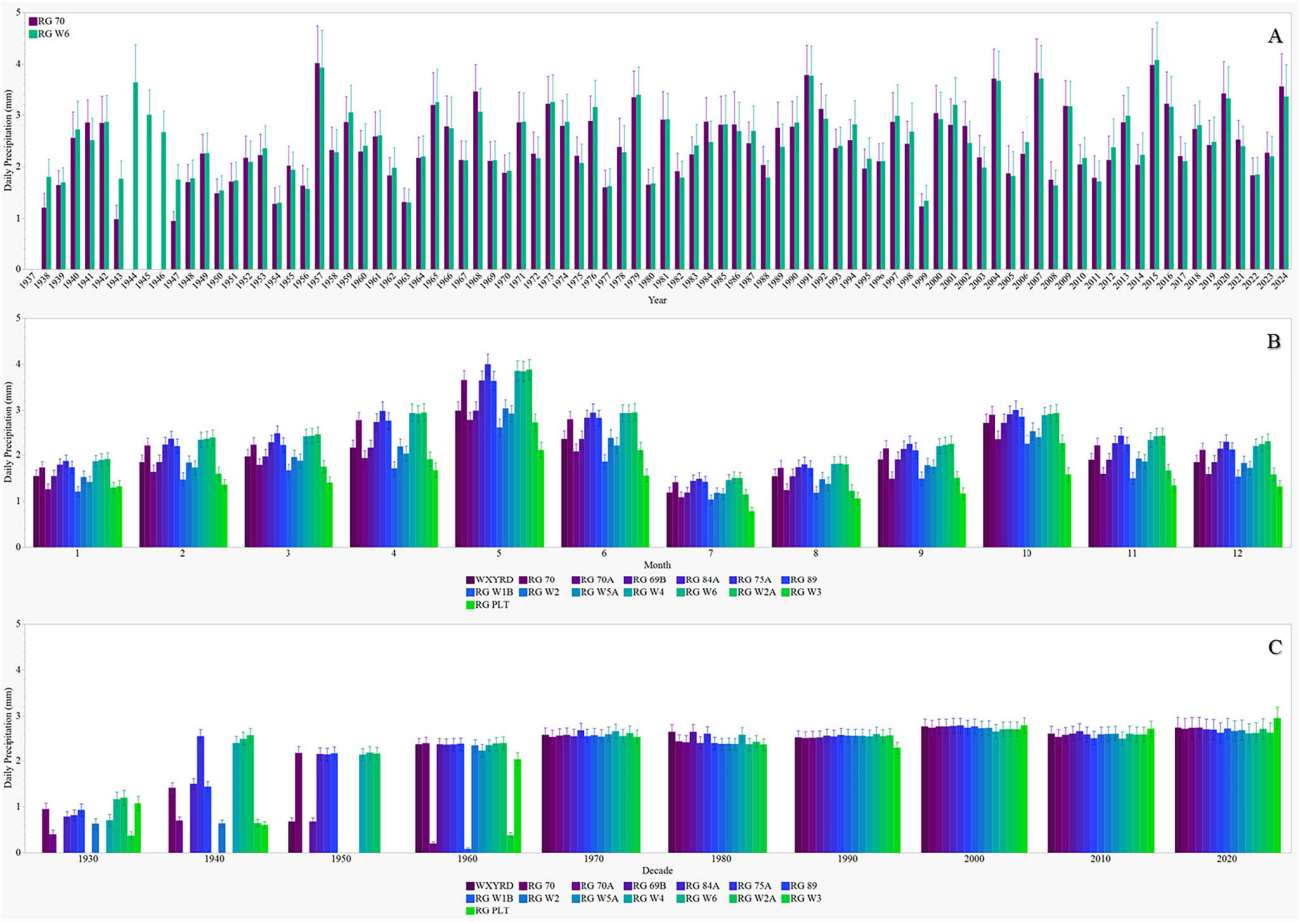
Average daily precipitation across years, months, and decades at the LTAR (Long‐Term Agroecosystem Research) Texas Gulf, Riesel, TX site. Panel A is the average daily precipitation (±1 SE) at two representative sites at the North and South ranch for the years 1937–2024. Panel B represents average daily precipitation for the month, while Panel C represents the average daily precipitation across the decade (±1 SE) at all 15 long‐term, on‐site rain gauge sites (1937–2024). From 1937 to 2001, precipitation was collected manually. In 2001, automated tipping buckets and dataloggers were used to measure precipitation at these sites. Manual checks continue to occur to ensure tipping bucket accuracy; see Section 2 for more information. Months 1–12 refer to January–December, respectively. RG, rain gauge; WXYRD, weather yard rain gauge station.

Climate in this region is continental with long, hot summers and mild winters (Figure 2). The growing season lasts on average from early March to mid‐November. Most precipitation occurs in spring and autumn, and convective thunderstorms during the summer months contribute to intense, short‐duration rainfall events. Tropical hurricanes can also produce substantial rainfall, but their occurrence is rare. Freezing rain, sleet, and snow occur occasionally but do not often contribute significant precipitation.

Agricultural production, environmental characteristics, and land use outcome monitoring have been occurring at this site for nearly 90 years. These data, in addition to periodic water quality, weather, lateral subsurface, shallow groundwater level, and land management data, are publicly available at https://www.ars.usda.gov/plains‐area/temple‐tx/grassland‐soil‐and‐water‐research‐laboratory/ .ars.usda.gov/plains‐area/temple‐tx/grassland‐soil‐and‐water‐research‐laboratory/. Installation of a VHF radio telemetry network was completed in 2001 to improve data quality and collection efficiency. From the on‐site base station, located at WXYRD (weather yard rain gauge station) (Figure 1), equipment maintenance and calibration are performed, and real‐time conditions are monitored. An automated data collection schedule runs continuously and collects data daily from each field station. Temperature data are currently being collected using a Campbell Scientific HMP45C‐L temperature and relative humidity probe (Campbell Sci 2026).

### 2.2 Historical precipitation

Historical precipitation data at the Riesel Watersheds, Texas Gulf LTAR site have been collected using network rain gauges (>1500 rain gauge years) at sub‐daily increments for most years from 1938 to the present (Harmel et al., 2014). On‐site historical precipitation data (i.e., station precipitation) used in this study were acquired from a suite of 15 rain gauges located in native prairie, row crop, and seeded pastureland sites on the Riesel Watershed study site (see Figure 1, Table 1). Manual precipitation rain gauges were used from 1938 to 2000 and from 2001 to the present, on‐site precipitation data at the Riesel Watershed site were collected using a CS‐700‐L Campbell Scientific tipping bucket and datalogger (Campbell Sci 2026). Because tipping bucket sensors have errors associated with high winds during storm events, manual rain gauges adjacent to automated sensors have been used to calibrate automated sensors since 2001.

**TABLE 1:** Rain gauge names, locations, and elevation.

Rain gauge name	Latitude	Longitude	Elevation, m (ft)
WXYRD	31.4778	−96.8853	175 (572)
RG70	31.4791	−96.8819	168 (548)
RG70A	31.4778	−96.8781	160 (523)
RG69B	31.4728	−96.8872	172 (564)
RG84A	31.4739	−96.8778	160 (524)
RG75A	31.4706	−96.8833	170 (555)
RG89	31.4672	−96.8825	172 (563)
RGW1B	31.4664	−96.8864	173 (567)
RGW2	31.4625	−96.8875	172 (561)
RGW2A	31.4625	−96.8819	169 (552)
RGW3	31.4631	−96.8797	172 (564)
RGW4	31.4567	−96.8881	169 (552)
RGW5A	31.4583	−96.8833	165 (540)
RGW6	31.4522	−96.8847	171 (560)
RG PLT	31.4561	−96.8708	157 (515)

Abbreviations: RG, rain gauge; WXYRD, weather yard rain gauge station.

Three gridded data sources were acquired for the exact latitude and longitude locations of each of the 15 rain gauges and compared to rain gauge data in this study (see Table 1): GridMET, DayMET, and PRISM.

GridMET is a high‐spatial resolution dataset of daily (∼4 km^2^) surface meteorological data covering the contiguous United States (CONUS) and British Columbia from 1979 to the present. It was created by combining attributes of two datasets: the North American Land Data Assimilation System Phase 2 (NLDAS‐2; Mitchell et al., 2004), and the PRISM (Daly et al., 2008) to better produce a comprehensive spatial and temporal dataset. Daily GridMET data can be accessed at https://app.climateengine.org/climateEngine (Abatzoglou, 2013).

DayMET provides daily estimates of gridded (∼1 km^2^) solar radiation, maximum and minimum temperature, precipitation, snow water equivalent, and water vapor over the CONUS, Hawaii, and Puerto Rico from January 1982 to December 2023. Local regression algorithms obtained from ground‐based surface meteorological stations are used to generate, interpolate, and extrapolate daily meteorological observations. Daily observed weather data of maximum and minimum temperature and precipitation used to generate DayMET data are obtained from GHCN‐Daily. A weighted linear regression‐based approach is used to consider the effect(s) of DayMET observations at varying elevations and to generate the daily meteorological variables for a particular grid cell. Surrounding weather stations to each grid cell are selected, and distance is weighted using a truncated Gaussian filter that considers the local station density. Daily DayMET data can be accessed at https://app.climateengine.org/climateEngineordaac.ornl.gov/cgi‐bin/dsviewer.pl?ds_id=2361 (Thornton & Devarakonda, 2011).

PRISM produces six essential climate parameters: precipitation, maximum and minimum temperature, dew point, and maximum and minimum vapor pressure. Station‐level climate data collected from ground‐based monitoring stations are used with various modeling techniques to generate daily precipitation data for a ∼800 km^2^ grid scale from 1895 to the present. PRISM uses a weighted linear regression approach with in situ station data to generate gridded climatic variables at varying elevations but also explicitly accounts for environmental variables such as terrain‐induced climate transitions, the effects of terrain as barriers, cold air drainage and inversions, and coastal effects. Daily PRISM data can be accessed at https://prism.oregonstate.edu/explorer (Daly et al., 2008).

### 2.3 Statistical analyses

All data in this study were analyzed using JMP pro (JMP statistical software 2026). Observed data from this study site included all days during the study period from 1982 to 2024 (15,702 rows of data) and only days with precipitation during the study period, where zero precipitation days were NOT included in the model (7567 rows of data). Multivariate correlation analyses and simple linear regression models were used to evaluate the relationships between the observed data collected at on‐site rain gauge stations and those collected from gridded datasets for all days (zero precipitation days included) and for the dataset that only included precipitation days (zero precipitation days NOT included). While observed historical rain gauge precipitation data from the years 1939–2024 are displayed in Figure 1 for all days (zero precipitation days included), multivariate correlation and regression analyses were limited to the years where gridded data were available (i.e., 1982–2024). Data from the three gridded data sources were also spatially matched via the nearest grid node to gauge locations using latitude–longitude coordinates, which, depending on the data source, could vary by up to 4 km (Table 1). To assess broader temporal patterns in precipitation, we calculated the daily average precipitation across all 15 rain‐gauge sites and the corresponding gridded values from GridMET, DayMET, and PRISM, which we refer to as *Average* in corresponding tables and figures. For this analysis, both observed datasets, which (1) included zero precipitation days and (2) did NOT include zero precipitation days, were first evaluated for skewness, but precipitation data did not follow a normal distribution given that precipitation is inherently skewed with greater numbers of low precipitation events. Consequently, no transformation affected skewness. However, we also tested the skewness in the residual data of both datasets and found that residuals were normally distributed, which, according to analysis of variance (ANOVA) rules, allows the data to be processed using linear analysis models with no transformations (Fernandez, 1992). Consequently, no transformations were made on either observed dataset, and all data from both datasets (as evaluated separately) were included in regression and multivariate correlation models.

Multivariate correlation analyses included a pairwise correlation where gauge‐measured precipitation and the corresponding daily values from GridMET, DayMET, and PRISM were computed for both datasets (i.e., [1] with zero precipitation days and [2] where zero precipitation days were NOT included).

Regression analyses were also evaluated for each gauge–gridded dataset pair, with gauge precipitation as the dependent variable and gridded precipitation as the predictor for both datasets (i.e., [1] with zero precipitation days and [2] where zero precipitation days were NOT included). Regression coefficients, coefficient of determination ($R^{2}$), residual patterns, and model diagnostics were used to assess the accuracy and bias of each gridded product relative to ground observations. For impact, we have selected the most useful dataset (i.e., either [1] where zero precipitation days were included or [2] where zero precipitation days were NOT included) to display within the manuscript depending on the research question.

To evaluate annual, seasonal, and daily patterns in precipitation, we used both a regression and ANOVA model from the *Average* data collected at rain gauge and gridded sites. Decades were sorted as follows: 1980 (1982–1989), 1990 (1990–1999), 2000 (2000–2009), 2010 (2010–2019), and 2020 (2020–2024). Seasons were defined using standard climatological groupings (winter: January–March; spring: April–June; summer: July–September; fall: October–December). To quantify the accuracy of each gridded climate dataset relative to on‐site observations, we first used a regression model to evaluate the relationship between rain gauge and gridded data sources by year (a continuous variable) and decade (a categorical variable) and another regression model to evaluate the relationship between the residual error of the gridded data estimates to actual rain gauge data by decade (a categorical variable) for the *Average* of these 15 locations. Residual error is defined as the difference between the observed daily precipitation at each rain gauge location (*y*) and the predicted precipitation by each gridded dataset (DayMET, GridMET, and PRISM) (*ŷ*), expressed *e *= *y* − *ŷ*. For this analysis, data were used that included all days, including zero precipitation days, and we conducted a secondary analysis of only days when precipitation occurred. A mixed model ANOVA analysis was further used to evaluate differences in rain gauge and gridded data by decade, season, and the interaction of decade and season when decade and season were set as categorical variables. For this analysis, we only used the second dataset where zero precipitation days were NOT included in the dataset so to focus solely on days with precipitation. Mean comparisons among decades, seasons, and the interaction between decade and season were further clarified using Tukey's honestly significant difference (HSD), where different letters indicate differing means.

Finally, we used both a regression and a one‐way ANOVA analysis to compare differences between actual rain gauge measurements and gridded precipitation data during extreme daily events or daily precipitation events that yielded 10% of the total average annual precipitation for this site (i.e., ≥90 mm day^−1^). Per this definition, only 20 observations during the 42 years were included in the analysis, and there were no days without precipitation in the dataset. A regression analysis was first conducted on the *Average* data with gauge precipitation as the dependent variable and gridded precipitation as the predictors, where zeros were included in the *Average* calculation. Regression coefficients, the coefficient of determination ($R^{2}$), residual patterns, and model diagnostics were then used to assess the accuracy and bias of each gridded product relative to ground observations. A one‐way ANOVA model was then used to evaluate the differences in extreme precipitation events from the precipitation data sources by the independent effects of year, decade, and season. This analysis also focused solely on the *Average* of rain gauge and gridded data. Mean comparisons among years, decades, and seasons by either rain gauge or gridded data source were determined using Tukey's HSD, where different letters indicate differences among these variables.

## 3 RESULTS

### 3.1 Historical precipitation patterns

From 1938 to 2024, annual precipitation at this site averaged 908.3 mm (Figure 2). Among months, precipitation was greater in spring and fall with average daily amounts of 2.7 and 2.2 mm day^−1^, respectively, and least in summer months with an average of only 1.6 mm day^−1^ (Figure 2B). There was high variation in precipitation totals across the rain gauge network by site, month, decade, and season (Figure 2A–C, S1). Specifically, rain gauges 70A, W1B, W3, W5A, and PLT reported less monthly precipitation compared to 70, 84A, 75A, 89, W6, W4, and W2A, but these differences generally equalized across decades (Figure 2C). Air temperature showed an average daily maximum of 25.7°C and minimum of 13.6°C, with maximum temperatures as high as 47.1°C and minimum temperatures as low as −18.3°C (Figure S2A). Among months and seasons, temperature followed a unimodal distribution with the highest temperatures in the summer months at high and low averages of 31.2°C and 22.7°C, and least in the winter at high and low averages of 17.9°C and 5.6°C (Figure S2B).

### 3.2 Spatial correlations of ground‐based vs. gridded precipitation sources

Regression analyses between historical on‐site precipitation data and gridded databases from 1982 to 2024 or from when gridded data first became fully available indicate a significant relationship between site precipitation data at all 15 rain gauge sites and the gridded data derived from GridMET, DayMET, and PRISM (Tables 2 and 3; *p *< 0.0001). The best‐fitting regression between station and gridded data when all 42 years were included in the analysis was GridMET where the *R* ^2^ = 0.80 when averaged across all 15 rain gauge sites (Table 1, *p *< 0.0001). DayMET was also a well‐fitting model with an *R* ^2 ^= 0.65, followed by PRISM with an *R* ^2^ of 0.47 when all 15 sites were averaged. Individual stations varied in their correlations with gridded data quality, where DayMET had the greatest correlation at RGW3 with an *R* ^2^ of 0.64 and was least at WXYRD with *R* ^2 ^= 0.32. GridMET also had the greatest *R* ^2^ at RGW3 where *R* ^2 ^= 0.79, but was least at RG70 at *R* ^2 ^= 0.55. PRISM, alternatively, had lower variation in *R* ^2^ values but was greatest at RGW1B at *R* ^2 ^= 0.47 and least at RG PLT at *R* ^2 ^= 0.37. Individual stations had greater variation in both DayMET and GridMET compared to PRISM.

**TABLE 2:** Annual *R* ^2^ correlations among the rain gauge sites and gridded precipitation data sites from 1982 to 2024.

	WXYRD	RG70	RG70A	RG69B	RG84A	RG75A	RG89	RGW1B	RGW2	RGW2A	RGW3	RGW4	RGW5A	RGW6	RG PLT	Average
DayMET	0.324	0.436	0.624	0.618	0.626	0.552	0.563	0.636	0.638	0.627	0.639	0.632	0.631	0.621	0.545	0.653
GridMET	0.726	0.552	0.775	0.726	0.783	0.774	0.785	0.775	0.782	0.778	0.787	0.781	0.785	0.776	0.666	0.804
PRISM	0.416	0.456	0.453	0.415	0.457	0.459	0.456	0.468	0.466	0.466	0.462	0.454	0.458	0.463	0.374	0.465

*Note*: All days, including zero precipitation days, were included in the regression analysis. Rain gauge names are referred to along the *y*‐axis and include an average or a regression of the daily data from all rain gauge and gridded data sites across all years. All data were significantly different from 0 at *p* < 0.0001.

Abbreviations: DayMET, Daily Surface Weather and Climatological Summaries; GridMET, Gridded Surface Meteorological Dataset; PRISM, Parameter‐Elevation Regressions on Independent Slopes Model; RG, rain gauge; WXYRD, weather yard rain gauge station.

**TABLE 3:** Seasonal *R* ^2^ correlations among the rain gauge sites and gridded precipitation data sites from 1982 to 2024.

		WXYRD	RG70	RG70A	RG69B	RG84A	RG75A	RG89	RGW1B	RGW2	RGW2A	RGW3	RGW4	RGW5A	RGW6	RG PLT	Average
*Fall*	DayMET	0.258	0.440	0.675	0.661	0.681	0.564	0.588	0.680	0.681	0.675	0.690	0.671	0.682	0.644	0.636	0.688
	GridMET	0.769	0.633	0.820	0.770	0.833	0.813	0.836	0.827	0.830	0.823	0.841	0.817	0.833	0.801	0.722	0.846
	PRISM	0.431	0.444	0.439	0.430	0.439	0.436	0.436	0.442	0.440	0.447	0.436	0.434	0.432	0.455	0.347	0.445
*Spring*	DayMET	0.338	0.421	0.616	0.608	0.612	0.516	0.517	0.628	0.622	0.611	0.620	0.612	0.606	0.596	0.488	0.634
	GridMET	0.695	0.493	0.745	0.698	0.751	0.754	0.750	0.729	0.737	0.745	0.749	0.748	0.749	0.743	0.650	0.772
	PRISM	0.415	0.452	0.447	0.416	0.446	0.445	0.443	0.471	0.470	0.452	0.447	0.444	0.442	0.450	0.363	0.455
*Summer*	DayMET	0.423	0.473	0.533	0.565	0.535	0.478	0.489	0.563	0.568	0.563	0.560	0.568	0.571	0.562	0.456	0.591
	GridMET	0.713	0.468	0.740	0.707	0.740	0.720	0.753	0.752	0.754	0.750	0.742	0.751	0.750	0.749	0.650	0.770
	PRISM	0.345	0.432	0.422	0.343	0.439	0.460	0.449	0.448	0.446	0.457	0.458	0.454	0.459	0.446	0.372	0.451
*Winter*	DayMET	0.290	0.408	0.650	0.621	0.658	0.657	0.666	0.650	0.663	0.641	0.664	0.663	0.651	0.674	0.584	0.683
	GridMET	0.726	0.609	0.794	0.726	0.809	0.800	0.803	0.798	0.811	0.795	0.814	0.808	0.807	0.814	0.629	0.827
	PRISM	0.453	0.495	0.502	0.453	0.506	0.498	0.501	0.509	0.504	0.514	0.514	0.486	0.507	0.503	0.423	0.512

*Note*: All days, including zero precipitation days, were included in the regression analysis. Rain gauge names are referred to along the *y*‐axis and include an average or a regression of the daily data from all rain gauge and gridded data sites across all years. All data were significant at *p* < 0.0001.

Abbreviations: DayMET, Daily Surface Weather and Climatological Summaries; GridMET, Gridded Surface Meteorological Dataset; PRISM, Parameter‐Elevation Regressions on Independent Slopes Model; RG, rain gauge; WXYRD, weather yard rain gauge station.

When evaluated across seasons, both DayMET and GridMET had the greatest correlations in fall at *R* ^2 ^= 0.69 and 0.85, respectively, and were least in summer at *R* ^2^ of only 0.59 and 0.77, respectively (Table 3, *p *< 0.0001). Alternatively, PRISM had the greatest correlations in winter at *R* ^2 ^= 0.51 and was least in fall at *R* ^2 ^= 0.45. The relationships between individual rain gauge station sites and gridded data also varied by season. For example, both DayMET and GridMET data sources had the greatest correlation in fall at RGW3 at *R* ^2 ^= 0.69 and 0.84, respectively, while DayMET showed the least correlation at WXYRD in fall at only *R* ^2 ^= 0.26, and GridMET had the least correlation in summer at RG70 at *R* ^2 ^= 0.47. PRISM, alternatively, had the greatest correlations with the individual rain gauge stations RGW2A and RGW3 in winter where both had *R* ^2 ^= 0.51, and had the least correlation at WXYRD with *R* ^2 ^= 0.35.

### 3.3 Temporal correlations of ground‐based vs. gridded precipitation sources

#### 3.3.1 Temporal regression and residual differences

**FIGURE 3:**
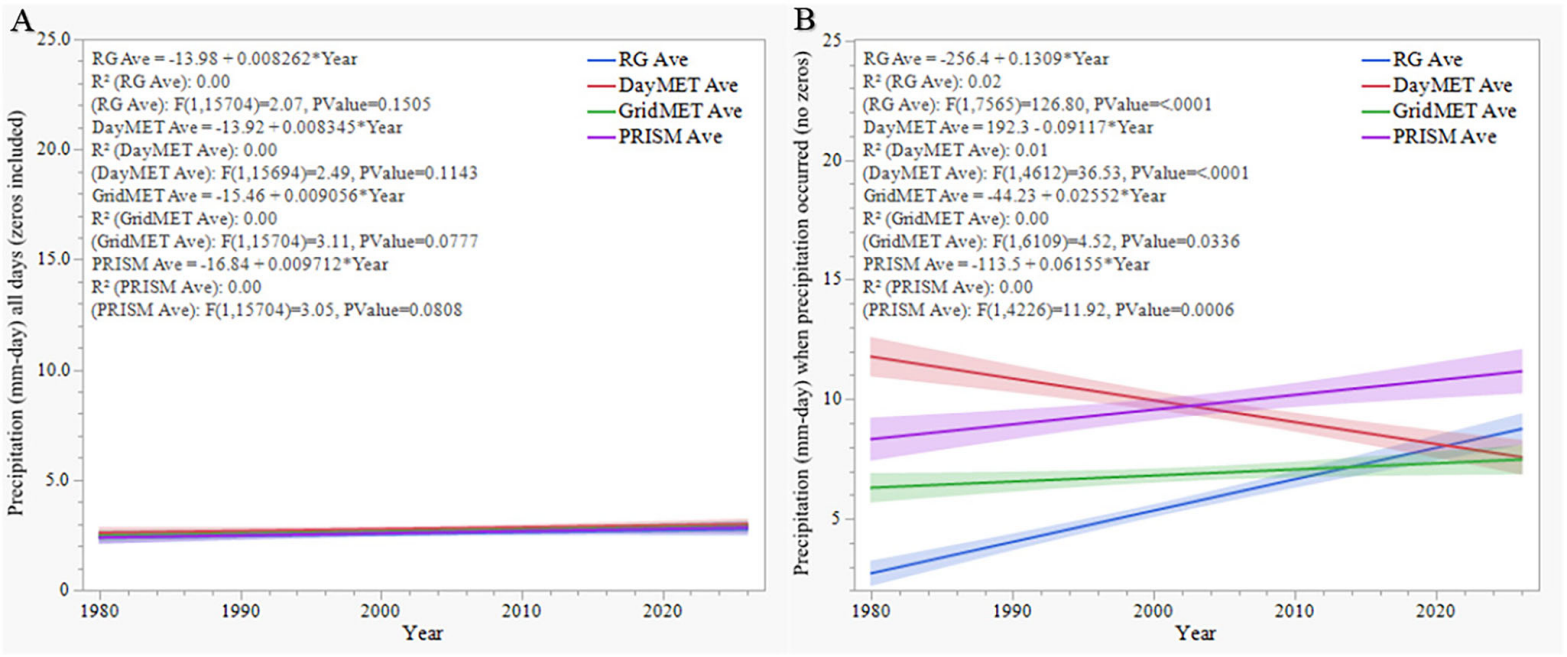
Regression relationships between precipitation data sources and year during all days. Panel A refers to days, including when precipitation did not occur, in the model while Panel B refers to only days when precipitation events occurred, zero precipitation days NOT included in the model. Models are considered significant if *p *< 0.05.

Regression analysis comparing precipitation abundance from the *Average* of all precipitation data sources through time indicated that there was no increase in precipitation across the 42 years of the study period (Figure 3A, Figure S3A), which was the case when the entire observed rain gauge dataset was included in the analysis, that is, zero precipitation days included. When zero precipitation days were NOT included in the analysis, there was a significant increase in precipitation abundance by ∼1–4 mm day^−1^ though time as measured by the rain gauge, GridMET, and PRISM data (Figure 3B, Figure S3B). DayMET, alternatively, showed a significant decrease by ∼6 mm day^−1^ in precipitation from 1982 to 2024.

**FIGURE 4:**
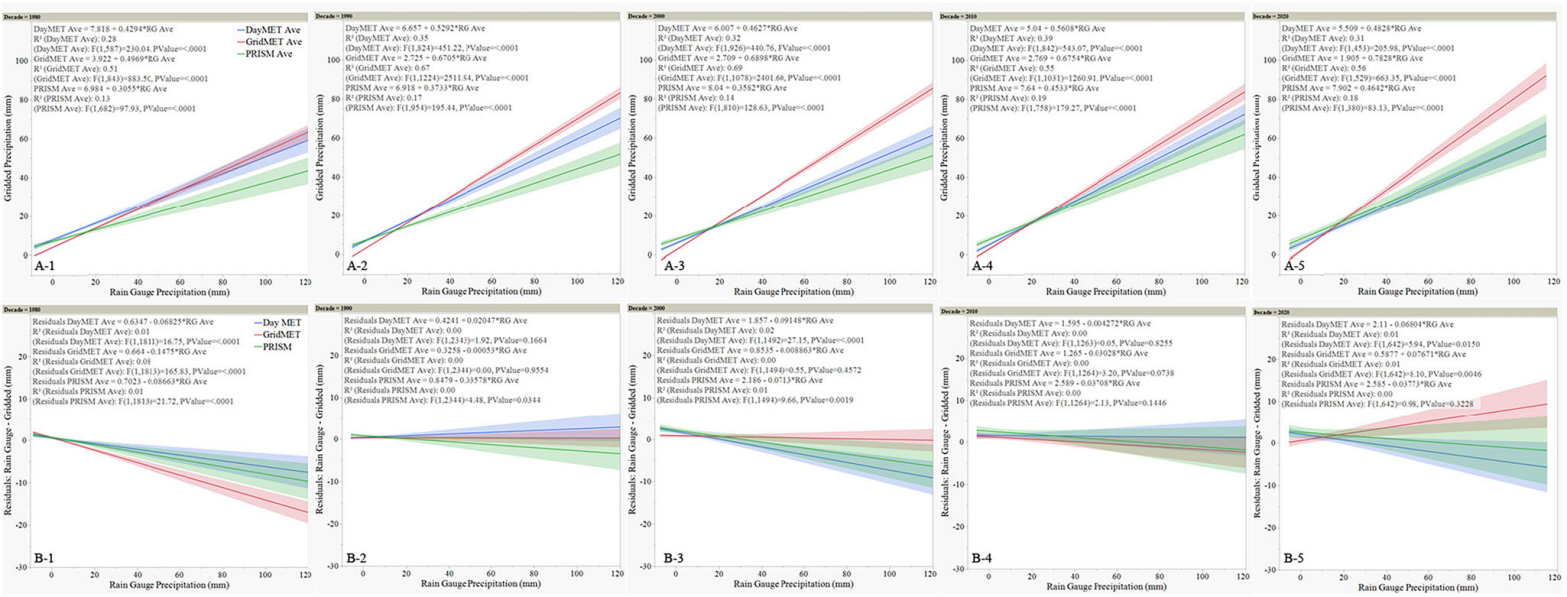
Regressions (A) and residual error (B) of the *Average* daily precipitation (mm) from the three gridded climate datasets compared to the 15 on‐site rain gauges sorted by decade during precipitation days where zero precipitation days were NOT included in the analysis (1980–2024; Figures 1, 2, 3, 4, 5).

The regression analysis for days with precipitation (i.e., zero precipitation days NOT included in the analysis) indicated that the *R* ^2^ values between gridded and rain gauge data improved between the 1980 and 1990 decades and remained relatively steady until the end of the study period (2024) (Figure 4A, Figure S4A, *p *< 0.0001). From 1982 to 1989, for example, the average *R* ^2^ for GridMET, DayMET, and PRISM were 0.51, 0.28, and 0.13, respectively (Figure 4A1). From 1990 to 2020, the *R* ^2^ of GridMET ranged from 0.55 to 0.67, DayMET ranged from 0.31 to 0.39, and PRISM ranged from 0.14 to 019 (Figure 4A2–A5).

Regressions of residual error between the *Average* precipitation at the 15 rain gauge sites and the three gridded data sources when precipitation occurred (i.e., zero precipitation days NOT included in the analysis) indicated that error was greatest in the 1980s, where all three gridded datasets underestimated large rainfall abundances and overestimated small rainfall totals (Figure 4B, Figure S4B; *p *< 0.05). When daily precipitation abundances were ≥120 mm day^−1^ during the 1980s decade, DayMET, GridMET, and PRISM underestimated precipitation by an average of 5, 15, and 7 mm day^−1^, respectively (Figure 4B1). By 1990, data from all three gridded data sources match site‐level rain gauge data values (Figure 4B2). In the 2000s, both DayMET and PRISM underestimated rain gauge precipitation by approximately 10 mm when precipitation exceeded 120 mm day^−1^, while GridMET did not differ from rain gauge observations (Figure 4B3). In the 2010s, all three gridded data sources matched site‐level rain gauge data (Figure 4B4). By 2020, PRISM accurately approximated rain gauge data, while GridMET overestimated precipitation by ∼10 mm and PRISM underestimated precipitation by ∼5 mm when precipitation ≥120 mm day^−1^ (Figure 4B5).

#### 3.3.2 Temporal analysis of variance results

Precipitation differed among decades and seasons and by the interaction among decade and season as evaluated via ANOVA models using only data from when precipitation occurred (zero precipitation data NOT included in the analysis) and from the *Average* of the 15‐rain gauge and co‐located gridded data sites (Table 4, Figure S4). Among decades, for example, actual precipitation from the rain gauges indicates that mean precipitation abundance nearly doubled following the 200's compared to the 1980s and 1990s from an average of 3.8 mm day^−1^ during precipitation events from 1982 to 2000 to 7.3 mm day^−1^ from 2000 to 2024 during precipitation events (Table 4A). Gridded data sources indicate varying results. For example, there were no differences among decades for GridMET, while DayMET precipitation averages were greater in 1980 and 1990 at average daily events amounts of 10.8 mm day^−1^ compared to the 2000–2020 decades where precipitation averaged 8.7 mm day^−1^ for each precipitation event. Alternatively, PRISM data indicate that when precipitation events occurred, there was less precipitation in each event in the 1980s compared to 2000–2020 where the average precipitation for daily events was 8.3 mm day^−1^ for 1980 but was 10.4 mm day^−1^ for the 2000–2020 decades.

**TABLE 4:** Mixed‐model analysis of variance (ANOVA) results of the differences in precipitation abundances during all precipitation events (zero precipitation days NOT included in the analysis) among decades and seasons from the average data collected at rain gauge and gridded sites.

A: Decade	Rain gauge (*p *< 0.001)		DayMET (*p *< 0.001)		GridMET (*p *> 0.05)		PRISM (*p *< 0.001)	
	LS mean (± SE)	MC	LS mean (± SE)	MC	LS mean (± SE)	MC	LS mean (± SE)	MC
1980	3.7 (0.3)	b	10.6 (0.5)	a	6.3 (0.4)	NS	8.3 (0.5)	b
1990	3.9 (0.3)	b	10.9 (0.4)	a	6.6 (0.3)	NS	9.1 (0.4)	ab
2000	6.7 (0.3)	a	9.6 (0.4)	ab	6.8 (0.3)	NS	10.2 (0.5)	a
2010	7.5 (0.3)	a	8.5 (0.4)	b	7.2 (0.3)	NS	10.3 (0.5)	a
2020	7.7 (0.5)	a	8.0 (0.5)	b	7.6 (0.4)	NS	10.7 (0.7)	a

B: Season	Rain gauge (*p *< 0.001)		DayMET (*p *< 0.001)		GridMET (*p *< 0.001)		PRISM (*p *< 0.001)	
	LS mean (± SE)	MC	LS mean (± SE)	MC	LS mean (± SE)	MC	LS mean (± SE)	MC
Fall	6.0 (0.3)	b	10.1 (0.4)	a	7.5 (0.3)	a	10.2 (0.5)	a
Spring	7.2 (0.3)	a	10.7 (0.4)	a	8.0 (0.3)	a	11.4 (0.4)	a
Summer	5.7 (0.4)	bc	9.3 (0.5)	ab	6.0 (0.3)	b	8.8 (0.5)	b
Winter	4.8 (0.3)	c	8.0 (0.4)	b	6.3 (0.3)	b	8.5 (0.5)	b

C: Decade × Season	Rain gauge (*p *= 0.0198)		DayMET (*p *> 0.05)		GridMET (*p *> 0.05)		PRISM (*p *> 0.05)	
	LS mean (± SE)	MC	LS mean (± SE)	MC	LS mean (± SE)	MC	LS mean (± SE)	MC
1980, Fall	4.6 (0.6)	cdefg	11.8 (0.9)	NS	7.4 (0.7)	NS	9.2 (0.9)	NS
1980, Spring	4.4 (0.6)	cdefg	11.7 (0.9)	NS	7.0 (0.6)	NS	9.5 (1.0)	NS
1980, Summer	2.6 (0.6)	g	9.7 (1.1)	NS	5.0 (0.8)	NS	7.3 (1.2)	NS
1980, Winter	3.1 (0.6)	fg	9.1 (1.0)	NS	5.8 (0.7)	NS	7.2 (1.0)	NS
1990, Fall	4.3 (0.5)	cdefg	12.2 (0.8)	NS	7.9 (0.6)	NS	10.3 (0.9)	NS
1990, Spring	4.2 (0.5)	defg	10.9 (0.8)	NS	6.8 (0.6)	NS	9.3 (0.9)	NS
1990, Summer	3.2 (0.5)	fg	10.6 (0.9)	NS	5.5 (0.6)	NS	8.4 (1.0)	NS
1990, Winter	4.0 (0.5)	efg	9.8 (0.8)	NS	6.3 (0.6)	NS	8.5 (0.8)	NS
2000, Fall	7.0 (0.6)	bc	10.0 (0.7)	NS	7.7 (0.6)	NS	10.8 (0.9)	NS
2000, Spring	7.8 (0.6)	ab	11.3 (0.8)	NS	7.7 (0.6)	NS	12.0 (0.9)	NS
2000, Summer	6.6 (0.7)	bcde	9.1 (0.9)	NS	5.2 (0.6)	NS	9.4 (1.0)	NS
2000, Winter	5.5 (0.6)	bcdefg	8.0 (0.7)	NS	6.6 (0.6)	NS	8.7 (0.9)	NS
2010, Fall	8.0 (0.7)	ab	9.0 (0.7)	NS	7.7 (0.6)	NS	11.2 (0.9)	NS
2010, Spring	8.5 (0.6)	ab	9.3 (0.7)	NS	7.9 (0.6)	NS	11.5 (0.9)	NS
2010, Summer	7.5 (0.8)	ab	8.3 (0.9)	NS	6.0 (0.7)	NS	8.8 (1.0)	NS
2010, Winter	6.0 (0.6)	bcdef	7.3 (0.7)	NS	7.1 (0.6)	NS	9.8 (0.9)	NS
2020, Fall	6.1 (1.0)	bcdefg	7.3 (1.0)	NS	6.8 (0.9)	NS	9.7 (1.4)	NS
2020, Spring	10.9 (0.9)	a	10.1 (0.9)	NS	10.2 (0.8)	NS	14.7 (1.2)	NS
2020, Summer	8.4 (1.1)	abcd	8.9 (1.2)	NS	7.8 (1.0)	NS	9.9 (1.5)	NS
2020, Winter	5.3 (0.9)	bcdefg	5.8 (0.9)	NS	5.5 (0.8)	NS	8.3 (1.3)	NS

*Note*: Table A refers to decade, B to season, and C to the interaction of decade and season. LS means refer to mean comparisons in mm precipitation among decades, seasons, and their interaction, while different letters indicate Tukey's honestly significant difference (HSD) mean differences. NS refers to a nonsignificant interaction.

Abbreviations: DayMET, Daily Surface Weather and Climatological Summaries; GridMET, Gridded Surface Meteorological Dataset; MC, mean comparison; PRISM, Parameter‐Elevation Regressions on Independent Slopes Model.

Seasonally, when zero precipitation days were NOT included in the analysis, spring had the greatest precipitation abundance among all precipitation data sources with an average of 7.2 mm day^−1^ at rain gauge sites, 10.5 mm day^−1^ for DayMET, 7.7 mm day^−1^ for GridMET, and 11.0 mm day^−1^ for PRISM during precipitation events. Winter, alternatively, was generally found to have the least precipitation among the evaluated data sources, with only 4.8 mm day^−1^ at the rain gauge sites, 8.0 mm day^−1^ for DayMET, 6.4 mm day^−1^ for GridMET, and 8.6 mm day^−1^ for PRISM during precipitation events. GridMET, however, identified that summer had less precipitation at this site compared to winter, at 5.7 mm day^−1^ during summer and at 6.4 mm day^−1^ during winter when precipitation occurred.

The interaction of seasons and decades when zero precipitation days were NOT included in the analysis only indicated that rain gauge data varied by this interaction (Table 3C). Among decades and seasons, for example, precipitation abundance was greatest in the 2020 decade during spring and was least in the 1980 decade during summer.

### 3.4 Daily rainfall distribution patterns

**FIGURE 5:**
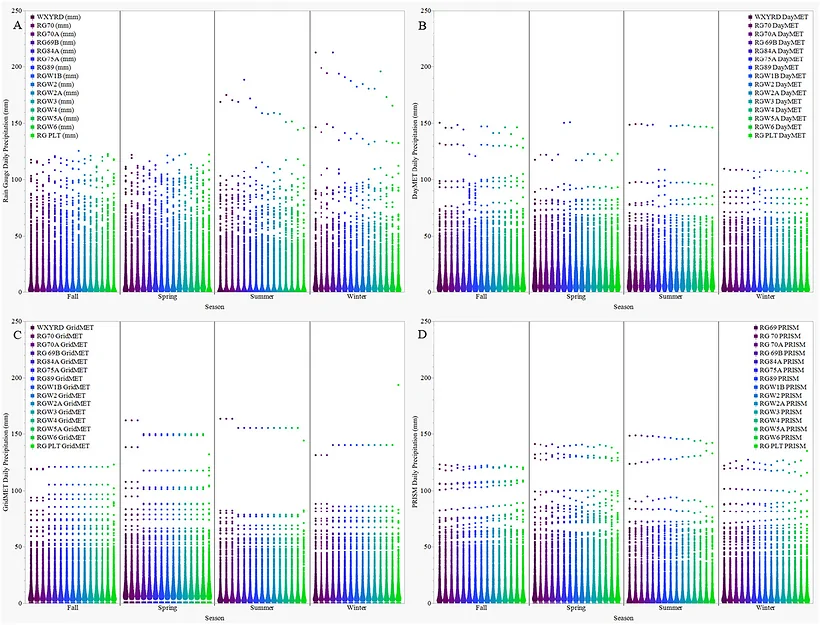
Distribution of total daily precipitation (mm) with all days (zero days included) in the figure, at all 15 sites among weather data sources by season. Panel A refers to on‐site rain gauge data, Panel B refers to DayMET (Daily Surface Weather and Climatological Summaries), Panel C refers to GridMET (Gridded Surface Meteorological Dataset), and Panel D represents PRISM (Parameter‐Elevation Regressions on Independent Slopes Model). RG, rain gauge; WXYRD, weather yard rain gauge station.

When all data including zero precipitation events were included in a model, daily precipitation among all 15 rain gauge sites and for all data sources across seasons demonstrates a similar precipitation pattern as was shown in monthly data Figure 2B, where winter and summer had less precipitation compared to spring and fall (Figure 5). Across winter and summer seasons, for example, a north‐to‐south gradient in precipitation abundance was observed among rain gauge locations (Figure 5A). These patterns were, however, not captured using any of the evaluated gridded data sources. Alternatively, both GridMET and PRISM did specify greater numbers of heavy precipitation events in both summer and winter (Figure 5C,D). All three gridded data sources showed a few heavy daily precipitation events in spring and summer, and DayMET identified a greater number of daily precipitation events in fall (Figure 5B–D). Unlike the other gridded data sources, DayMET did not indicate any heavy daily precipitation events in winter (Figure 5B).

Among years for the average of the 15 rain gauge sites, the only significant differences in extreme precipitation abundance, where precipitation was ≥90 mm day^−1^, across years and decades occurred in the rain gauge sites (Table 5, Figure 5). Furthermore, this difference only occurred between the 1980 compared to the 1990–2020 decades and was likely due to the single high precipitation event (174.4 mm day^−1^) that occurred in 1986 at the rain gauge sites. This single high precipitation event yielded a greater average for the 1980s decade at 174.4 mm day^−1^ compared to the 1990–2020 decades, where averages ranged from 101.2 to 112.3 mm day^−1^. This high precipitation event in 1986 was also found to be significantly greater than most other extreme precipitation events except for summer 2001, summer 2005, winter 2007, and spring (*p *= 0.03, Table 5). ANOVA results did not identify any seasonal differences in the number of precipitation events or precipitation abundance amounts among any of the data sources evaluated for the extreme precipitation events. However, more frequent extreme events were observed in spring and fall (*n* = 13) compared to winter and summer (*n* = 7). A visual observation also identified three times as many extreme precipitation events in the last 24 years 2000–2024 (*n* = 15) compared to the first 18 years (*n *= 5) of this 42‐year study period (Figure 5, Table 5).

**TABLE 5:** Years and total precipitation (mm) for each daily precipitation event ≥90 mm as measured by average of all on‐site rain gauges (RG) or from the average of all gridded data collected at these sites including DayMET (DM [Daily Surface Weather and Climatological Summaries]), GridMET (GM [Gridded Surface Meteorological Dataset]), or PRISM (P [Parameter‐Elevation Regressions on Independent Slopes Model]).

	Fall				Spring				Summer				Winter			
Year	RG	DM	GM	P	RG	DM	GM	P	RG	DM	GM	P	RG	DM	GM	P
1986	–	–	–	–	–	–	–	–	–	–	–	–	174.3	84.9	72.1	66.2
1991	95.7	96.2	92.1	107.1	–	–	–	–	–	–	–	–	–	–	–	–
1997	117.1	125.3	96.2	106.9	–	–	–	–	–	–	–	–	–	–	–	–
1997	–	–	–	–	100.2	34.9	58.2	50.0	–	–	–	–	–	–	–	–
1998	91.6	100.0	85.2	103.7	–	–	–	–	–	–	–	–	–	–	–	–
2000	97.5	41.9	74.0	45.8	–	–	–	–	–	–	–	–	–	–	–	–
2001	–	–	–	–	–	–	–	–	101.5	50.8	55.4	56.4	–	–	–	–
2004	–	–	–	–	94.6	55.1	72.8	128.6	–	–	–	–	–	–	–	–
2005	–	–	–	–	–	–	–	–	149.8	52.5	54.9	128.7	–	–	–	–
2007	–	–	–	–	–	–	–	–	–	–	–	–	118.1	79.7	54.0	117.7
2013	–	–	–	–	–	–	–	–	–	–	–	–	92.1	42.5	81.6	81.4
2015	–	–	–	–	93.1	17.5	44.9	60.9	–	–	–	–	–	–	–	–
2015	115.9	169.1	112.9	121.4	–	–	–	–	–	–	–	–	–	–	–	–
2015	103.6	86.2	98.9	119.8	–	–	–	–	–	–	–	–	–	–	–	–
2016	–	–	–	–	–	–	–	–	–	–	–	–	100.9	58.2	133.5	125.5
2016	–	–	–	–	110.6	63.9	137.8	139.1	–	–	–	–	–	–	–	–
2019	–	–	–	–	100.5	74.7	82.8	82.1	–	–	–	–	–	–	–	–
2020	–	–	–	–	–	–	–	–	94.1	133.9	144.0	145.9	–	–	–	–
2024	–	–	–	–	109.7	81.9	138.3	130.9	–	–	–	–	–	–	–	–
2024	–	–	–	–	104.4	73.1	108.3	99.6	–	–	–	–	–	–	–	–

*Note*: Colors refer to the decade.

During intense precipitation events when precipitation ≥90 mm day^−1^, there were no significant relationships between rain gauge and gridded data sources (*p *> 0.05, Figure 5, Figures S5 and S6). For these extreme events, root mean squared error (RMSE) ranged from a low average of 28.45 mm day^−1^ for GridMET to a high average of 53.27 mm day^−1^ for PRISM. Moreover, there was greater error between gridded and rain gauge data during winter (± 108.1 mm), and summer (±97.3 mm) compared to fall (±53.2 mm) and spring (±46.7 mm) (Table 6). In winter, for example, DayMET underestimated rain gauge precipitation by 89.4 mm in 1986, by 38.4 mm in 2007, by 49.6 mm in 2013, and by 42.7 mm in 2016 when precipitation was ≥90 mm day^−1^. GridMET data were also poorly representative of most extreme precipitation events ≥90 mm day^−1^ during winter, where precipitation was underestimated by 102.2 mm in 1986 and by 64.1 mm in 2007, was only underestimated by 10.5 mm in 2013, but was overestimated by 12.6 mm in 2016. Winter precipitation events of ≥90 mm day^−1^ were more accurately represented with PRISM data, but during 1986, extreme precipitation events were underestimated with PRISM by 108.1 mm, by only 0.4 mm and 10.7 mm in 2007 and 2013, respectively, and PRISM overestimated precipitation by 24.6 mm in 2016. During summer, the discrepancy between rain gauge and gridded data varied when precipitation ≥90 mm day^−1^, where in 2001, DayMET, GridMET, and PRISM all underestimated rain gauge precipitation by 50.7, 46.1, and 45.1 mm, respectively. In 2004, DayMET, GridMET, and PRISM again underestimated rain gauge precipitation but by 97.3, 94.9, and 21.1 mm, respectively. Alternatively, by 2020, when the average of the 15 rain gauge sites was 94.1 mm, gridded precipitation of DayMET, GridMET, and PRISM overestimated precipitation by 39.8, 49.9, and 51.1 mm, respectively. During fall, the error between average rain gauge and average gridded dataset precipitation ranged from 0.5 to 55.6 mm for DayMET, 3.0 to 23.5 mm for GridMET, and from 5.5 to 51.7 mm for PRISM when precipitation events ≥90 mm day^−1^. Similarly, spring precipitation error between rain gauge and gridded data indicated that DayMET overestimated precipitation by 25.8–65.3 mm, GridMET data varied from rain gauge data by 3.9–48.2 mm, and PRISM ranged from 4.8 to 50.2 mm different than measured rain gauge data for precipitation events ≥90 mm day^−1^.

**TABLE 6:** Analysis of variance (ANOVA) of the relationships between year, decade, and season for extreme daily precipitation events of ≥90 mm day^−1^.

	Rain Gauge (*p *= 0.0313)		DayMet (*p *> 0.05)		GridMET (*p *> 0.05)		PRISM (*p *> 0.05)	
Year	LS mean	MC	LS mean	MC	LS mean	MC	LS mean	MC
1986	174.4	a	84.9	NS	72.1	NS	14.6	NS
1991	95.7	b	96.2	NS	92.1	NS	15.7	NS
1997	108.7	b	80.1	NS	77.2	NS	29.0	NS
1998	91.6	b	100.0	NS	85.2	NS	11.0	NS
2000	97.5	b	42.0	NS	74.0	NS	45.8	NS
2001	101.5	ab	50.8	NS	55.4	NS	16.3	NS
2004	94.7	b	30.2	NS	44.3	NS	7.2	NS
2005	149.8	ab	21.2	NS	54.9	NS	128.7	NS
2007	118.1	ab	59.9	NS	52.5	NS	18.1	NS
2013	92.1	b	42.5	NS	81.6	NS	81.4	NS
2015	104.2	b	50.5	NS	65.8	NS	60.5	NS
2016	105.8	b	61.1	NS	27.6	NS	132.3	NS
2019	100.5	ab	74.7	NS	82.8	NS	7.7	NS
2020	94.1	b	133.9	NS	59.5	NS	145.9	NS
2024	107.1	b	23.9	NS	117.2	NS	18.9	NS

	Rain gauge (*p *= 0.0039)		DayMET (*p *> 0.05)		GridMET (*p *> 0.05)		PRISM (*p *> 0.05)	
Decade	LS mean	MC	LS mean	MC	LS mean	MC	LS mean	MC
1980	174.4	a	84.9	NS	72.1	NS	14.6	NS
1990	101.2	b	89.1	NS	82.9	NS	21.2	NS
2000	112.3	b	40.8	NS	56.2	NS	43.2	NS
2010	102.4	b	55.8	NS	59.6	NS	76.4	NS
2020	102.8	b	60.5	NS	97.9	NS	61.3	NS

	Rain gauge (*p *> 0.05)		DayMET (*p *> 0.05)		GridMET (*p *> 0.05)		PRISM (*p *> 0.05)	
Season	LS mean	MC	LS mean	MC	LS mean	MC	LS mean	MC
Fall	103.6	NS	82.9	NS	83.3	NS	33.5	NS
Spring	101.9	NS	38.4	NS	68.1	NS	43.3	NS
Summer	115.2	NS	68.6	NS	56.6	NS	97.0	NS
Winter	121.4	NS	61.4	NS	62.4	NS	59.9	NS

*Note*: Tables A refers to Year, B to Decade, and C to the Season. LS means refer to mean comparisons, and different letters indicate mean differences as evaluated using Tukey's honestly significant difference (HSD). NS refers to a nonsignificant interaction.

Abbreviations: DayMET, Daily Surface Weather and Climatological Summaries; GridMET, Gridded Surface Meteorological Dataset; MC, mean comparison; PRISM, Parameter‐Elevation Regressions on Independent Slopes Model.

## 4 DISCUSSION

### 4.1 Historical precipitation patterns

Historical precipitation patterns across nearly 90 years of precipitation data collected at this 340 ha (840 acre) site in the southern Great Plains and Texas Gulf region indicate the large variability across both space and time. Annual precipitation varied by more than 1200 mm year^−1^ (Figure 2A), with many seasons of deluge and/or drought as reported in multiple previously published reports from this ecoregion (i.e., Flanagan & Mahmood, 2023; Harmel et al., 2003). While the trend in drought and deluge was continuous throughout the years, an increase in precipitation in the decades following 2000 was likely due to upgraded and more sensitive instrumentation that was installed in 2001 (Figure 3, Table 4A; Harmel et al., 2007, 2014). A surprising finding was the high variation in monthly precipitation abundance across the rain gauge network, as this network spans less than a 7.75 km^2^ (3 mi^2^) area (Figure 2B). As each of these rain gauges is quality controlled, the differences among rain gauge locations are likely due to common storm patterns and how each storm intersected with the topography at each rain gauge location. In topographically diverse regions, Henn et al. (2018) suggest that precipitation can differ by more than 200 mm year^−1^. This area, however, is not topographically complex as this region has common prairie features such as rolling hills with little variation in elevation, as shown in Table 1. Convective storm patterns, however, vary spatially, especially in the Great Plains, with greater intense storm activities occurring at night and in the month of July (Reif & Bluestein, 2017). Moreover, Bluestein (2008) describes that even small topographical variation affects storm severity at a location. Consequently, the location of rain gauges matters even in simple topographical regions.

### 4.2 Gridded data assessments

Gridded precipitation datasets have, since the mid‐2000s, provided spatiotemporally complete precipitation data for models and management decisions at scale (Abatzoglou, 2013; Allen et al., 2021; Daly et al., 2008; Thornton et al., 2014). Like the model developers for each of these gridded precipitation data sources, we also found that on‐site precipitation at the Texas Gulf LTAR site was significantly correlated with historic gridded estimates. This is an important finding as quality assurance tests in rural or agricultural regions are rare. Quality assurance is key to reducing modeling errors, providing informed adaptive management recommendations, and improving forecasting skill. The three gridded datasets in this study were chosen because they are commonly used across multiple agroecosystem modeling applications such as the Agricultural Policy/Environmental Extender Model https://epicapex.tamu.edu/apex/ and the rangelands analysis platform https://rangelands.app/ (Robinson et al., 2019; Williams & Izaurralde, 2010). Gridded precipitation data are also used in determining insurance policies and premiums for producers, especially relating to the Pasture, Range and Forage Insurance programs (Campiche & Jones, 2013).

In support of our first hypothesis, we identified that these three gridded data sources varied in their accuracy compared to rain gauge data. Interestingly, we also found that grid scale did not appear to affect data accuracy. Instead, GridMET (4 × 4 km grid scale) data were more closely aligned with our ground‐based station precipitation data than DayMET (1 × 1 km grid scale) or PRISM (0.8 × 0.8 km grid scale) for the 42 years of this study (Table 2). In a case study in the US corn belt, Mourtzinis et al. (2017) determined that DayMET precipitation had greater correlations with ground‐monitoring data than PRISM, but they also found that RMSE was greater with DayMET compared to PRISM. Ray et al. (2022), alternatively, identified that PRISM had higher correlations and lower RMSE than DayMET across a Texas watershed. The differences in these authors’ results may be due to different methods associated with tipping bucket precipitation sensors used at ground‐based monitoring sites. This is largely because tipping bucket rain gauges often produce underestimates of precipitation, especially at high rainfall intensities, if not calibrated with manually monitored rain gauges (Shedekar et al., 2016). Calibration and quality control are also particularly important for regions that receive frequent convective storms (Segovia‐Cardozo et al., 2023). Given that the Texas Gulf LTAR rain gauge network is quality controlled via manual rain gauges for when convective storms occur and continually monitored, these data are likely more representative of actual precipitation abundances and serve as a strong representative of gridded precipitation dynamics (Harmel et al., 2003). Consequently, it appears that GridMET currently provides the most accurate estimates of gridded precipitation across this region.

In support of our second hypothesis, we found that while all correlations between gridded and rain gauge data were significant, the goodness of fit varied by years and seasons during the 42‐year study period (Tables 2 and 3). Across years, gridded data accuracy improved through time, where gridded data collected prior to 2000 had greater mean squared error and poorer correlations compared to data collected after 2000 (Figures 3 and 4, Table 4). A primary reason for higher data accuracy through time is likely due to improved instrumentation at weather stations in recent years (Sose & Sayyad, 2016). Seasonally, we identified the greatest correlation between rain gauge and gridded data during fall and winter compared to spring and summer (Table 3). However, the seasonal accuracy of gridded meteorological products when compared to on‐site monitoring stations may vary by area of interest. Xu et al. (2019), for example, indicated that rain gauge precipitation had greater correlations with gridded precipitation in the northern Great Plains of south‐central Canada and northern Montana/North Dakota during fall and spring compared to summer and winter. Differences in gridded data seasonal accuracy may be due to extreme storm events that commonly occur in the summer and winter seasons of the northern Great Plains as compared to the extreme storm events that occur in the southern Great Plains during spring and summer and the spatial variation of these storms across these differing ecoregions (Song et al., 2019; Verevkin & Folkins, 2023).

Extreme daily precipitation events have been argued to be increasing in the southern Great Plains (Easterling et al., 2000). However, more recent and local analyses indicated that, while temperature shifts have been occurring in the Texas High Plains, particularly due to increasing minimum temperatures, there were no differences in precipitation dynamics across 207 weather stations and 0.25° grid points across a 52‐year study period (Long et al., 2012). Moreover, both Harmel et al. (2003) and Flanagan & Mahmood (2023) indicated that extreme precipitation events only increased during winter. Our results do indicate the presence of extreme daily precipitation events in winter, but we found no indication of increasing frequency or intensity of these events annually or seasonally (Figure 5, Tables 5 and 6). Furthermore, in support of our third hypothesis, we found poor correlations between rain gauge and gridded data during extreme precipitation events when precipitation ≥90 mm day^−1^ (Figure 5, Figures S5 and S6, Table 6). Seasonally, for example, there was greater error between rain gauge data and gridded data observations in both winter and summer compared to fall and spring (Tables 4 and 5). It is possible that when intense storms bring ≥90 mm day^−1^ of precipitation during summer and winter, they are more localized compared to those that occur during fall and spring months. Winter and summer storm events in this region are also commonly due to El Niño Southern Oscillation (ENSO) conditions (Dai & Wigley, 2000). While a post hoc analysis of ENSO conditions over time suggests that there is no strong evidence indicating a change in ENSO over the 42 study years at this site (Figures S5 and S6, *p *< 0.0001), when ENSO does occur, it does produce intense and localized storm events that contribute to heavy precipitation (Goddard & Gershunov, 2020; Lepore et al., 2017). Consequently, while intense storms are occurring in this area, there does not appear to be an increase in these events during the years analyzed. However, because of the localization of storm events, using gridded data to understand intense storms is likely not practical given the high error between site and gridded measurements.

Currently, multiple agroecosystem models use gridded weather and climate data to make continental estimates and predictions on water quality, agroecosystem management, and spatiotemporal forecasting (Abbott et al., 1986; Arnold et al., 2012; Bergström, 1995; Hartman et al., 2020; Rigge et al., 2021; Robinson et al., 2019; Schantz et al., 2025, 2026). Given that precipitation timing, intensity, and abundance are key factors affecting model output, a more comprehensive assessment of weather data sources is necessary (Chen et al., 2019; Huxman et al., 2004; Murphy et al., 2015; Waller et al., 2021). Multiple authors have worked to summarize the benefits and drawbacks of weather data types including gridded or ground‐based weather station datasets as well as satellite, radar, or remotely sensed data (Blankenau et al., 2020; Essou et al., 2017; Mankin et al., 2025; Ray et al., 2022). Collectively, multiple authors appear to agree that ground‐based weather and climate data are superior to gridded or satellite precipitation data for point‐scale measurements (Mankin et al., 2025; Ray et al., 2022). These papers all conclude, however, that the poor distribution of ground‐based monitoring stations requires more data availability and thus improved abilities to acquire data in rural or topographically complex regions, which is possible when using satellite, remotely sensed, or radar‐based measurements (Gebremichael, 2010; Kober & Tafferner, 2009; Louf et al., 2019; Sun et al., 2016; Thies and Bendix, 2011). Given that ground‐based weather monitoring station density is currently decreasing (Sun et al., 2016) and that instrumentation of satellite, remotely sensed, or radar‐based products is improving (Sose & Sayyad, 2016), it seems plausible that the most accurate precipitation data sources will soon be those that integrate ground‐based measurements with satellite, remotely sensed, or radar‐based products. Consequently, accuracy assessments of weather and climate data sources for the use of agroecosystem management are important to continue, especially across spatiotemporal scales.

## 5 CONCLUSION

This study quantified the spatiotemporal accuracy of three common gridded precipitation products: GridMET, DayMET, and PRISM using an independent 42‐year record from a dense 15‐gauge network at the Texas Gulf LTAR site. Quality assurance tests of gridded precipitation data are important to evaluate in rural and agriculturally important regions. Because of the nearly 90‐year precipitation record at the Texas Gulf LTAR site in Riesel, TX, we were able to comprehensively evaluate the differences in precipitation timing and abundance of three commonly used gridded weather and climate data sources, that is, GridMET, DayMET, and PRISM. While precipitation in this area is highly variable both spatially and temporally, our findings suggest that gridded data had as much as 80% correlation rates with on‐site rain gauge precipitation abundances and that GridMET was more aligned with ground‐based weather station precipitation data, both annually and seasonally. Another key finding of this study was that gridded data calculated from earlier decades (1982–2000) had greater error compared to recent decades (2000–2024), which is likely due to upgraded instrumentation at ground‐based sites used to produce gridded datasets. No gridded data source, however, accurately accounted for daily extreme precipitation, regardless of season of the year. Based on these findings, using this information for risk management or mitigation models relating to erosion or crop insurance claims will likely result in inaccurate results and is not recommended. Future studies should continue to test the quality of precipitation data sources, especially as instrumentation improves and because greater reliance on satellite, radar, or remotely sensed products is likely. This study, however, provides a template for continuing quality assurance tests of gridded precipitation data sources across spatiotemporal scales, which can be applied across the 19 continental US LTAR network sites.

## AUTHOR CONTRIBUTIONS

**Merilynn C. Hirsch‐Schantz**: Conceptualization; formal analysis; writing—original draft. **Douglas R. Smith**: Project administration; writing—review and editing. **R. Daren Harmel**: Conceptualization; writing—review and editing. **Kabindra Adhikari**: Formal analysis; writing—review and editing. **Kelly R. Thorp**: Writing—review and editing. **Stuart P. Hardegree**: Conceptualization; methodology. **Andrew Fullhart**: Writing—review and editing. **John T. Abatzoglou**: Resources. **Javier M. Osorio Leyton**: Writing—review and editing. **Roger Sheley**: Writing—review and editing. **Aaron Hird**: Project administration; resources.

## CONFLICT OF INTEREST STATEMENT

The authors declare no conflicts of interest.

## Supporting information



Supplementary Material
