# Supplementary material for: Accuracy assessments of gridded precipitation data: A case study from LTAR Texas Gulf
Source: J Environ Qual. 2026 Aug 20;55(4):e70236. doi: 10.1002/jeq2.70236 (PMC13494067; doi:10.1002/jeq2.70236)
Supplement: Supplementary file 1 [file JEQ2-55-0-s001.docx]

Supplementary Material/Appendix

**Figure S1.** Mean precipitation ± standard error of daily precipitation events mm day^-1^ for the *Average* of the 15 rain gauge sites and the co-located data derived from DayMET, GridMET and PRISM by decade and season for the study period (1982-2024). Differences were observed for the independent effects of decade and season for all data sources, but the interaction of decades and seasons were only significant for rain gauge data (see Table 4).

B

A

**Figure S2:** Figure A and B refer to the annual (A) and monthly (B) temperatures as collected from the WXYRD on-site monitoring station. Months 1-12 refer to Jan-Dec, respectively.

B

A

**Figure S3.** Regression relationships between precipitation data sources and year during all days with all data included. Figure A refers to days, including when precipitation did not occur, in the model while Figure B refers to only days when precipitation events occurred, or no zeros in the model. Models are considered significant if *p*<0.05. This figure is similar to Figure 3, but includes data points for additional information.

B-5

B-4

B-3

B-2

B-1

A-1

A-2

A-3

A-4

A-5

A-1

A-2

A-3

A-4

A-5

A-5

A-4

A-3

A-2

A-1

**Figure S4:** Regressions (A) and residual error (B) of the *Average* daily precipitation (mm) from the three gridded climate datasets compared to the 15 on-site rain gauges sorted by decade during precipitation days where zero precipitation days were NOT included in the analysis (1980-2024; Figures 1-5). Figure is similar to Figure 4, but also includes data points for additional understanding.

**Figure S5.** Regression relationships between gridded and rain gauge precipitation during extreme precipitation events of ≥90 mm day^-1^.

**Figure S6.** Regression relationships between precipitation data sources and year during extreme precipitation events of ≥90 mm day^-1^.

**El Niño Southern Oscillation Methods**

El Niño Southern Oscillation (ENSO) events were accounted for in this study by first obtaining ENSO data from the multivariate ENSO Index Version 2 rank (MEI. v2) found at https://psl.noaa.gov/enso/mei/ (Wolter and Timlin 1998, Zhang et al. 2019). The multivariate index is calculated using 6 variables including sea-level pressure, zonal and meridional components of the surface wind, sea surface temperature, surface air temperature, and total cloudiness fraction of the sky (Wolter and Timlin 1993). The Multivariate ENSO Index Version 2 (MEI. v2) expands upon this initial assessment by calculating a time series of conditions over the tropic Pacific Basin using five of these variables including sea level pressure, sea surface temperature, surface zonal winds, surface meridional winds, and outgoing longwave radiation to produce a bi-monthly time series of ENSO conditions from 1979 to present and reduce interseason variability (Kobayashi et al. 2015, Zhang et al. 2019). These data include a quantitative value on the intensity of an El Niño or La Niña event which ranges from -3 to 3 where -3 refers to a very strong La Niña event while 3 refers to a very strong El Niño event (Wolter and Timlin 1998, Zhang et al. 2019).

A distribution model of ENSO MEI. v2 rank was first used to evaluate ENSO conditions at the study site. We then used an ANOVA model to evaluate how ENSO MEI. v2 rank varied among the 43 years of this study. Lastly, we used a regression model to compare the differing historical precipitation data sources to the ENSO MEI. v2 rank.

**El Niño Southern Oscillation Results and Discussion**

There was no strong evidence that ENSO events changed over the 43 study years at this site (Figure 4; *p*<0.0001). In general, there was a common sinewave pattern between El Niño and La Niña events across years, but there were more La Niña (N=267 months) compared to El Niño (N=249 months) events at this site (Figure S2). When El Niño events occurred, however, they tended to be stronger than La Niña where the greatest El Niño event was ranked at 2.64 out of a possible 3 and seven El Niño events were considered outliers when the distribution of events were analyzed (Figure S2). There were, alternatively, no La Niña events considered outliers, and the strongest La Niña event ranked at -1.8 out of a possible -3.

**Figure S4.** ANOVA of ENSO MEIv2 rank by year for the Texas Gulf LTAR site in Riesel, Texas. Colors indicate the ENSO category ranging from severe La Niña in blue to very severe El Niño in red (*p*<0.0001).

All historical precipitation data sources evaluated in this study showed positive correlations between precipitation and ENSO MEI v.2 rank (Figure 5, *p*<0.0001). La Niña events were often associated with lower precipitation compared to El Niño, and R^2^ values ranged from 0.025 for on-site station precipitation to 0.019 for PRISM data. Root mean squared error (RMSE) of these regressions were greatest at the ground-based weather station at 62.1 mm, was also high for both DayMET and GridMET at 61.5 mm and 61.5 mm, respectively, and least for PRISM with an RMSE of only 56.5 mm.

La Niña	El Niño

La Niña	El Niño

La Niña	El Niño

La Niña	El Niño

D

C

A

B

La Niña	El Niño

**Figure S5.** Historical monthly precipitation (mm) by ENSO MEI v.2 rank for the Texas Gulf LTAR site in Riesel, Texas. Independent figures represent historical precipitation data derived from gridded databases or on-site station data where Figure A represents gridMET, Figure B, DayMET, Figure C. PRISM, and Figure D represents the on-site weather station data. Colors indicate the ENSO category ranging from severe La Niña in blue to very severe El Niño in red. All models were significant at *p*<0.0001.

**Citations**:

Cui, W., X. Dong, B. Xi, and A. Kennedy. 2017. Evaluation of reanalyzed precipitation variability and trends using the gridded gauge-based analysis over the CONUS. *Journal of Hydrometeorology* 18:2227-2248.

Dai, A., and T. Wigley. 2000. Global patterns of ENSO‐induced precipitation. *Geophysical Research Letters* 27:1283-1286.

Denton, E. M., R. L. Sheley, and S. P. Hardegree. 2024. Spring precipitation amount and timing predict restoration success in a semi‐arid ecosystem. *Journal of Applied Ecology* 61:2985-2996.

Essou, G. R. C., R. Arsenault, and F. P. Brissette. 2016. Comparison of climate datasets for lumped hydrological modeling over the continental United States. *Journal of Hydrology* 537:334–345.

Hoover, D. L., W. K. Lauenroth, D. G. Milchunas, L. M. Porensky, D. J. Augustine, and J. D. Derner. 2021. Sensitivity of productivity to precipitation amount and pattern varies by topographic position in a semiarid grassland. *Ecosphere* 12:e03376.

Keener, V., G. Feyereisen, U. Lall, J. Jones, D. Bosch, and R. Lowrance. 2010. El-Niño/Southern Oscillation (ENSO) influences on monthly NO3 load and concentration, stream flow and precipitation in the Little River Watershed, Tifton, Georgia (GA). *Journal of Hydrology* 381:352-363.

Kobayashi, S., Ota, Y., Harada, Y., Ebita, A., Moriya, M., Onoda, H., Onogi, K., Kamahori, H., Kobayashi, C., Endo, H., Miyaoka, K., Takahashi, K., 2015. The JRA-55 Reanalysis: general specifications and basic characteristics. *Journal of the Meteorological Society of Japan* 93, 5-48. DOI: 10.2151/jmsj.2015-001

Krishnamurthy, L., G. A. Vecchi, R. Msadek, A. Wittenberg, T. L. Delworth, and F. Zeng. 2015. The seasonality of the Great Plains low-level jet and ENSO relationship. *Journal of Climate* 28:4525-4544.

Lindström, G., B. Johansson, M. Persson, M. Gardelin, S. Bergström. 1997. Development and test of the distributed HBV-96 hydrological model. *Journal of Hydrology.* 201: 272-288

Lipp, E. K., N. Schmidt, M. E. Luther, and J. B. Rose. 2001. Determing the effects of El Niño-Southern Oscillation events on coastal water quality. *Estuaries* 24:491-497.

Reif, D. W., and H. B. Bluestein. 2017. A 20-year climatology of nocturnal convection initiation over the central and southern Great Plains during the warm season. *Monthly Weather Review* 145:1615-1639.

Roy, T., X. He, P. Lin, H. E. Beck, C. Castro, and E. F. Wood. 2020. Global evaluation of seasonal precipitation and temperature forecasts from NMME. *Journal of Hydrometeorology* 21:2473-2486.

Stewart, I. D., and T. R. Oke. 2010. Thermal differentiation of local climate zones using temperature observations from urban and rural field sites. Ninth symposium on urban environment, Keystone, CO. p. 2-6.

Wolter, K., and M. S. Timlin, 1998: Measuring the strength of ENSO events - how does 1997/98 rank? *Weather,* **53,** 315-324. DOI: [10.1002/j.1477-8696.1998.tb06408.x](https://doi.org/10.1002/j.1477-8696.1998.tb06408.x).

Zhang, T, A. Hoell, J. Perlwitz, J. Eischeid, D. Murray, M. Hoerling and T. Hamill, 2019: Towards Probabilistic Multivariate ENSO Monitoring, *Geophysical Research Letters*, 46, DOI: [10.1029/2019GL083946](https://doi.org/10.1029/2019GL083946)
